# Cost sharing for breast cancer hormone therapy: How do dual eligible patients’ copayment impact adherence

**DOI:** 10.1371/journal.pone.0250967

**Published:** 2021-05-18

**Authors:** Siyu Ma, Donald S. Shepard, Grant A. Ritter, Robert E. Martell, Cindy Parks Thomas

**Affiliations:** 1 The Heller School for Social Policy and Management, Brandeis University, Waltham, MA, United States of America; 2 Tufts Medical Center, Tufts University, Boston, MA, United States of America; Keck School of Medicine of the University of Southern California, UNITED STATES

## Abstract

**Objective:**

To examine the different levels of copayment assistance and treatment adherence among Medicare and Medicaid dual eligible beneficiaries with breast cancer in the U.S.

**Research design:**

Propensity Score methodology was adopted to minimize potential selection bias from the nonrandom allocation of the treatment group (i.e., full Medicaid beneficiaries) and control group (i.e., Medicare Savings Programs [MSPs] beneficiaries). Longitudinal hierarchical model and Cox proportional-hazard model were adopted to examine patients’ adherence over their full five-year course of adjuvant hormone therapy.

**Results:**

Our study cohort consisted of 1,133 dual eligible beneficiaries diagnosed with hormone receptor-positive early stage breast cancer in years 2007 –mid 2009. About 80.5% of them received MSPs benefits, while the rest received full Medicaid benefits. On average for a standardized 30-day hormone therapy medication, full Medicaid beneficiaries spent $0.5-$2.0 and MSP beneficiaries spent $1.4-$4.8 in copayment. After adjusting for other factors, this copayment reduction wasn’t associated with a significantly better adherence. However, when the catastrophic coverage threshold was reached (copayments reduced to zero), significant improvement in adherence was found in both groups.

**Conclusions:**

Our study found that small amount of cost-sharing reduction did not affect Medicare and Medicaid dual eligible patients’ medication treatment adherence, however, the elimination of cost-sharing (even a minimal amount) was associated with improved adherence. Future legislative and advocacy efforts should be paid on eliminating cost sharing for dual eligibles, and possibly even a broader group of financially vulnerable patients.

## Introduction

Breast cancer has the highest incidence rate of all cancer types among the U.S. women [1]. Hormone receptor-positive (HR+) breast cancers take up 80% of total breast cancers, which can be treated with adjuvant hormone therapy (AHT) after surgery and chemotherapy to prolong survival and lower the risk of breast cancer recurrence [2]. Tamoxifen and aromatase inhibitors (AIs) are two major AHT medications. Tamoxifen is the standard AHT medication for premenopausal women, while AIs (i.e., anastrozole, letrozole, and exemestane) are the first AHT medicine choice for postmenopausal women [3]. Clinical practice guideline recommends that patients diagnosed with HR+ breast cancer to stay on their AHT for at least 5 years after treatment onset to achieve the best clinical outcomes [3]. However, adherence to AHT remains suboptimal [4]. First year AHT adherence among younger women (below 65 years old) was found to be between 72% to 81% [5–8], and it was even worse among older women (above 65 years old) [9].

About 20% of elderly Medicare beneficiaries (65 years and older) are also enrolled in Medicaid benefits due to low income and assets or disability. These “dual eligible” beneficiaries (i.e., Medicare and Medicaid) account for 34% of total Medicare spending, for they usually have costly healthcare needs [10]. Extra help (also known as Low Income Subsidy [LIS]) is a program that provides help to people with limited income and resources like dual eligible beneficiaries to pay their prescription drug costs (e.g., premiums, deductibles, and coinsurance). Dual eligible beneficiaries either receive full Medicaid benefits in addition to Medicare benefits (Full Medicare beneficiaries must have income and assets below $2,000 or $3,000 if married in 2019) or some who do not qualify for full benefits due to excess resources (i.e., higher income), receive benefits through Medicaid-administered Medicare Savings Programs (MSPs) (MSP beneficiaries must have income and assets below $7,730 or $11,600 if married in 2019) [11, 12]. In general, full Medicaid beneficiaries receive greater copayment assistance than those who receive benefits from one of the MSPs, but the differences are generally small (usually a few dollars difference in coinsurance for a given medication, plan premium and deductibles are waived for both groups). In addition, like all other Medicare Part D beneficiaries, when dual eligible beneficiaries spend over certain coinsurance amount and copayment within a year (i.e., $5,100 in 2019), they (both full Medicaid and MSPs beneficiaries) automatically enter the “catastrophic phase” where their out-of-pocket payment amount becomes zero.

Following enactment of the Modernization of Medicare Advantage Act, an increasing number of health insurance plans adopted the value-based insurance design (VBID) model and reduced or eliminated out-of-pocket costs (OOPCs) for selected medications for chronic conditions to improve adherence. Choudhry *et al*. found that Pitney Bowes’ (a large Fortune 500 company) copayments reduction and elimination policy was associated with an immediate adherence increase to medication treatment among its members, and the effect was maintained in the following year [13]. Maeng *et al*. examined the Geisinger Health Plan’s zero co-pay program and found that during the months when the program was in place, their eligible employees were more likely to fill drug prescriptions than non-eligible employees [14]. While the impact of cost-sharing on adherence is well documented, very limited studies focused such relationships among breast cancer treatment nor dual eligible beneficiaries, the most costly and also the poorest population in the U.S [15, 16]. It is a clear gap in the literature and it’s important to generate evidence on the dual eligible group because they account for a third of total Medicare spending [10].

The objective of this study was to examine the association between different levels of copayment assistance and AHT adherence among Medicare and Medicaid dual eligible beneficiaries with breast cancer over the full five-year course of treatment. We hypothesize that decreasing copayment by a small amount will have little to no effect on AHT adherence; however, eliminating cost-sharing for dual eligible beneficiaries will immensely increase their AHT adherence. Because previous studies on Medicaid beneficiaries showed that even minimal amount of out-of-pocket payment would reduce their likelihood to fill any prescription [17–19].

## Materials and methods

Brandeis University Institutional Review Board approval was obtained before the start of this study. This is a retrospective longitudinal cohort study to compare the overall AHT adherence between full Medicaid and MSP recipients among dual eligible beneficiaries. Adherence and persistence are two measures adopted to reflect different aspects of patients’ overall adherence to medication treatment. We used medication possession ratio (MPR) as the measure of patients’ adherence to medication treatment, which reflects the percentage of total insurance covered days that a patient had AIs on hand within a given period (one year in this study). Adherence measures how often patients take their medication per year, in other words, patients’ compliance in the context of ongoing use. Persistence, on the other hand, is a time-to-event measure of continued AI use. Non-persistence is defined as the first time the gap between two filled prescriptions of a patient was large enough to reflect likely discontinuation of treatment. More detailed constructions of the two measures are explained in dependent variables section.

### Data source

SEER- Medicare-linked database for the years of 2007–2014 is used for this study. SEER-Medicare data links two population-based datasets providing detailed information on Medicare beneficiaries with cancer. For this study, we extracted patient demographics, cancer diagnosis, time of diagnosis, and initial therapy (surgery and/or radiation) from the SEER component of the data [20], and information on Medicare enrollment, covered drugs, fill dates, and days of supply from the Medicare-based component of the data. All data were fully anonymized and the Brandeis IRB waived the requirement for informed consent.

### Study sample

Our study cohort is female “dual eligible” beneficiaries aged 65 years and older, diagnosed with hormone receptor positive early stage (stage I-III) breast cancer in years 2007-mid 2009. Among those who initiated AHT (at least one filled AI prescriptions) within the first year of breast cancer diagnose, inclusion criteria are to be continuously enrolled (with no gap greater than 45 days) in both Medicaid and in Medicare Parts A, B, and D from diagnosis till five years after the first filled AI prescription. Exclusion criteria include patients with: 1) unknown stage or HR status at diagnosis, 2) unknown or missing race value, 3) diagnosis of other cancers or more than one breast cancer, or 4) time in an inpatient facility for the entire year, 5) missing or switched extra help/LIS eligibility, or 6) unknown or missing dual eligible status.

### Variables

#### Dependent variables

Two study outcomes measure the impacts of cost-sharing on our two cohorts of patients: adherence and persistence. Adherence is constructed as the number of days of AI supplied divided by the number of days covered within a year. If a patient was dead in the previous year, he/she was excluded from the following year. Therefore, each patient could have at least 1 and up to 5 adherence time periods. As an alternative measure of adherence, we also constructed a dichotomous version using an MPR value of 80% or above as the threshold value (MPR> = 80% deemed as adherent) [9, 21–24].

We chose a gap of 90 days as non-persistence definition in our main analysis to be consistent with our adherence measure (a person with maximum of 73 days [20% of 365 days] without a prescription to be still considered adherent). This value might be considered a fairly stringent condition, thus we also performed sensitivity analysis using gaps of 180 days to see if the alternative greatly affected results [21, 22, 25]. Since our patients may remain AI persistent throughout their five years of treatment or die without being non-persistent, we rely on survival analysis, specifically Cox proportional hazard modeling, to address our research questions regarding persistence. As is common with survival analysis, each patient in the model has two outcome values: a time-to-event value reflecting first gap in filled prescriptions of 90 days or more, time to death, or five years of continued persistence, and a censor variable, valued at 1 for patients who died or reach five years without a gap of 90 days (i.e., censored), otherwise valued at 0 (i.e. not censored). In keeping with survival analytic methodology, the denominators used for the persistent rates include the number of patients still persistent at the start of each year. Patients already deemed non-persistent or dead in previous years are not included in following years.

### Covariates

Our treatment group is full Medicaid beneficiaries and control group is MSP beneficiaries. The number of months within a year that dual eligible beneficiaries were in the catastrophic phase denotes when they pay zero coinsurance or copay on drugs. Other covariates include age, race/ethnicity, marital status, income level (Zipcode level income), SEER registry area, metropolitan area, comorbidities (measured by Hierarchical Condition Category [HCC]: higher represents more comorbidities, and zero means no comorbidity), tumor characteristics (stage, size, lymph nodes involvement, grade), and treatment characteristics (i.e., surgery plus radiation vs no surgery). Number of months in the catastrophic phase, age, and comorbidities are time variant covariates, meaning each variable has at least 1 to 5 measures responding to year 1 to year 5 observation periods. A detailed definition of each covariate is provided in S1 Table.

#### Data analysis

Propensity Score methodology (PSM) was adopted to minimize potential selection bias from the nonrandom allocation of the treatment group, namely full Medicaid beneficiaries. Because our sample size was small, the kernel weighting approach was chosen as the method for implementing PSM with the bandwidth of 0.06 for optimizing the balance between variance and specificity [26]. Weights were calculated in such a way to provide an average treatment effect on the treated (ATT). Other PSM techniques (i.e. nearest neighbor matching) might further shrink our sample size or lessen the representativeness of the results. In order to avoid the issue that weighting method may give extremely high weights to certain outliers, we trim predicted weights at the 95 percentile and redistributed the excess weight to other members of the study sample. All variables described in the covariates section were included in the model to determine propensity score. Once kernel weights were determined, longitudinal hierarchical models (with repeated measures nested within patient) were used to examine adherence measure. These models were tested for collinearity, and final specifications had no variables with VIF over 2.5. Following our adherence modeling, we conducted a Cox proportional-hazard model to examine the effects of treatment and other covariates on the hazard rate for patient non-persistence. Two-sided test with an alpha of 0.05 deemed as statistical significance. All statistical analysis was conducted using SAS v9.3.

## Results

**Fig 1:**
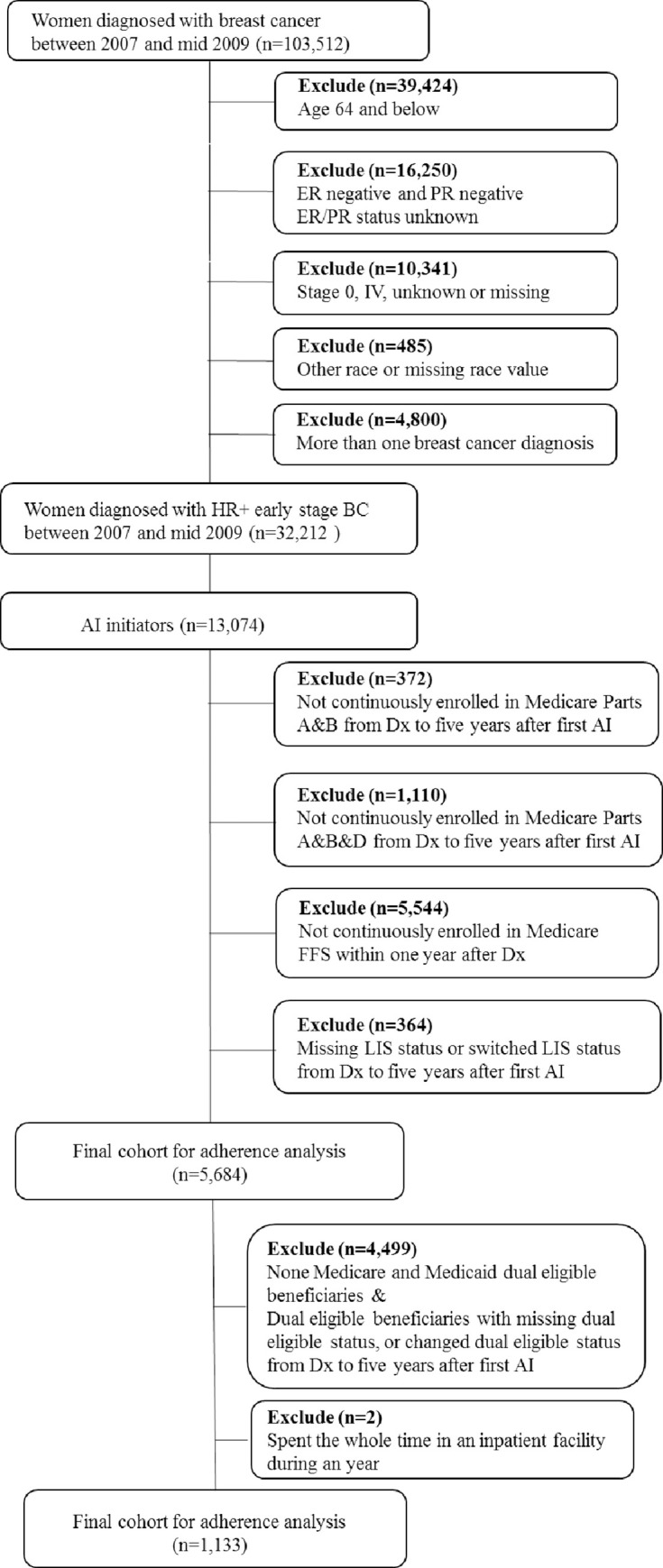
Selection criteria for identifying Medicare and Medicaid dual eligible beneficiaries diagnosed with hormone receptor-positive early stage breast cancer from 2007 to mid-2009.

Our final study cohort consisted of 1,133 dual eligible beneficiaries diagnosed with HR+ early stage breast cancer in years 2007 –mid 2009 (Fig 1). About 80.5% of these beneficiaries received full Medicaid benefits, while the rest received MSP benefits. On average, the full Medicaid beneficiaries spent $2.00 copays on 30-day standardized AIs in year 1 and the amount decreases over the five years, while MSP beneficiaries spent $4.77 copays on 30-day standardized AIs in year 1 and the amount also decreases throughout the years (S2 Table). Table 1 compares the baseline information of each the two groups. The mean age for full Medicaid beneficiaries was 75, while MSP beneficiaries were on average one year younger. Compared with MSPs beneficiaries, full Medicaid beneficiaries were more likely to be White (p<0.001), be married (p<0.05), live in metropolitan area (p<0.001), and have HCC scores, a relative risk score, of three or more (A score of three predicts healthcare costs three times that of an average Medicare beneficiary) (p<0.001). On the other hand, the full Medicaid and MSP groups had similar breast cancer tumor characteristics and treatment plans. After PSM, our treatment and control groups are well balanced: standardized variable differences are within the recommended limits of -0.25 and 0.25 (S1 Fig).

**Table 1:** Baseline characteristics of Medicare and Medicaid dual eligible beneficiaries diagnosed with hormone receptor-positive early stage breast cancer from 2007 to mid-2009, by the source of dual benefits.

Characteristics	Full Medicaid Benefits	MSP benefits	P^/^^a^
	n (%)	n (%)	
**Age Group, y**^**/**^^**b**^			0.091
65–69	60 (27.3)	247 (27.0)	
70–74	65 (29.5)	221 (24.1)	
75–79	51 (23.2)	193 (21.1)	
80+	44 (20.0)	254 (27.8)	
**Race/Ethnicity**			<0.001
White, non-Hispanic	169 (76.8)	484 (52.9)	
Non-White	51 (23.2)	431 (47.1)	
**Comorbidity (HCC score)** ^**/**^^**c**^			<0.001
0	54 (24.5)	177 (19.3)	
1	69 (31.4)	193 (21.1)	
2	40 (18.2)	186 (20.3)	
3+	57 (25.9)	359 (39.2)	
**Married**			0.028
Yes	36 (16.4)	212 (23.2)	
No	184 (83.6)	703 (76.8)	
**Income level**^**/**^^**d**^			<0.001
High	33 (15.6)	204 (23.2)	
Middle high	48 (22.6)	211 (20.8)	
Middle low	63 (29.7)	225 (25.6)	
Low	68 (32.1)	240 (27.3)	
**SEER Registry Region**			<0.001
Northeast	39 (17.7)	168 (18.4)	
South	165 (75.0)	174 (19.0)	
Midwest	5 (2.3)	77 (8.4)	
West	11 (5.0)	496 (54.2)	
**Metropolitan Area**			<0.001
Yes	147 (66.8)	798 (87.2)	
No	73 (33.2)	117 (12.8)	
**Tumor Stage**			0.902
I	102 (46.4)	421 (46.0)	
II	90 (40.9)	367 (40.1)	
III	28 (12.7)	127 (13.9)	
**Tumor Size**			0.268
< 1.0cm	102 (46.4)	421 (46.0)	
> = 1.0cm	90 (40.9)	367 (40.1)	
Unknown	28 (12.7)	127 (13.9)	
**Lymph Node Positivity**			0.074
0 (negative)	114 (51.8)	488 (53.3)	
> = 1	81 (36.8)	278 (30.4)	
Unknown	25 (11.4)	149 (16.3)	
**Tumor Grade**^**/**^^**e**^			0.233
Well differentiated	37 (16.8)	203 (22.2)	
Moderately differentiated	109 (49.5)	453 (49.5)	
Poorly differentiated	58 (26.4)	208 (22.7)	
Unknown	16 (7.3)	51 (5.6)	
**Treatment**			0.197
Surgery + radiation	87 (39.6)	367 (40.1)	
Surgery, no radiation	127 (57.7)	497 (54.3)	
No surgery	6 (2.7)	51 (5.6)	
**Total**^/^^f^	220	915	

Notes

a. *statistically significant at p<0.05 level, ** at p<0.01 level, *** at p<0.001 level; NS stands for not significant

b. average age for full Medicaid beneficiaries was 75 and for MSP beneficiaries was 74 (p = 0.016)

c. Comorbidity is constructed with the macro developed by NIH Healthcare Delivery Research Program. The macro is available to download here: https://healthcaredelivery.cancer.gov/seermedicare/considerations/charlson.comorbidity.macro.sas

d. Income level is divided based on quintiles of zipcode level median household annual income (High: > = $50,307; Middle high: $38,576-$50,307; Middle low: $30,520-$38,576; Low: <$30,520)

e. poorly differentiated tumors are more likely to grow and spread faster than well differentiated tumors, while moderately differentiated tumors are in between

f. full Medicaid beneficiaries took up 80.6% of total cohort while MSP beneficiaries stood for the rest of the 19.4% of the study cohort

### Adherence

Of the 1,133 dual eligible beneficiaries in our study sample, 904 (79.8%) were adherent (with MPR greater or equal to 80% defined as adherent, else non-adherent) during the first year that they filled their AI prescriptions. In the second to the fifth year of follow-up, the percent of patients who were adherent to their AI treatment since their first start dropped to 69.9%, 66.1%, 64.3%, and 53.8% respectively. During their third year of treatment, a higher percentage of full Medicaid beneficiaries were adherent to AHT compared with MSP beneficiaries (72.2% versus 64.7%, p<0.05). During the remaining follow-up periods, no statistically significant differences were found in HT adherence between treatment and control groups. Each year, MSP beneficiaries had slightly higher mean MPR than full Medicaid beneficiaries, but these unadjusted differences were not statistically significant (S3 Table).

In the adjusted model predicting adherence, we found that full Medicaid beneficiaries were not statistically significantly different from MSP beneficiaries. On the other hand, higher odds of adherence were associated with patients who spent more months in the catastrophic phase (OR: 1.933; p<0.001). The odds ratios predictions for covariates were shown in Table 2. Patients taking higher number of medications at the same time were associated with lower odds of being adherent (OR: 0.969, p<0.001). In addition, patients’ with high zipcode level income was estimated to have higher odds of adherence to HT compared to patients low zipcode level income (OR: 1.600; p<0.001). All other covariates were not statistically significantly associated with adherence. The results for the adjusted model predicting MPR can be found in S4 Table. The main findings mirrored the ones we found in the previous model. Full Medicaid and MSP beneficiaries didn’t have a significant difference in likelihood of being adherent. However, number of months a person in the catastrophic phase was significantly associated with MPR. Every one more month in the catastrophic phase led to 3.8% increase in MPR (P<0.001).

**Table 2:** Model-based adjusted odds ratio of predictors for aromatase inhibitor adherence measured by medication possession ratio (MPR) > = 80% among Medicare and Medicaid dual eligible beneficiaries diagnosed with hormone receptor-positive early stage breast cancer from 2007 to mid-2009.

Variable	Odds Ratio^/^^a^	95% CI	P
**Treatment and Control**			
Full Medicaid vs MSP	0.876	0.762 to 1.008	0.771
**Catastrophic Coverage Months**	1.933	1.693 to 2.207	<0.001
**Year**			
2 vs 1	0.406	0.283 to 0.583	<0.001
3 vs 1	0.458	0.317 to 0.660	<0.001
4 vs 1	0.568	0.389 to 0.830	<0.001
5 vs 1	0.341	0.231 to 0.505	<0.001
**Age, y**	1.004	0.986 to 1.023	0.210
**Race**			
Non-White vs White, non-Hispanic	1.088	0.978 to 1.212	0.272
**Comorbidity score**			
1 vs 0	1.036	0.772 to 1.391	0.283
2 vs 0	1.077	0.751 to 1.546	0.565
3+ vs 0	0.786	0.555 to 1.113	0.610
**Married**			
Yes vs No	0.747	0.544 to 1.026	0.691
**Income level**^**/**^^**b**^			
High vs Low	1.600	1.044 to 2.452	<0.001
Middle high vs Low	0.914	0.619 to 1.349	0.241
Middle low vs Low	0.882	0.594 to 1.308	0.452
**SEER Registry Region**			
Midwest vs West	1.071	0.494 to 2.215	0.626
Northeast vs West	0.786	0.385 to 1.607	0.430
South vs West	0.894	0.670 to 1.193	0.082
**Metropolitan Area**			
Yes vs No	0.894	0.670 to 1.193	0.876
**Tumor Stage**			
II vs I	1.191	0.825 to 1.719	0.113
III vs I	1.103	0.667 to 1.823	0.367
**Variable**	**Odds Ratio**	**95% CI**	**P**
**Lymph Node Positivity**			
> = 1 vs 0 (negative)	0.787	0.537 to 1.154	0.213
**Tumor Size**			
> = 1cm vs <1cm	1.197	0.872 to 1.644	0.961
**Tumor Grade**			
Moderately vs Well differentiated	1.458	1.061 to 2.003	0.738
Poorly vs Well differentiated	1.070	0.748 to 1.532	0.480
**Treatment**			
Surgery + radiation vs No surgery	1.581	0.733 to 3.408	0.216
Surgery, no radiation vs No surgery	1.491	0.701 to 3.169	0.275
**Number of Medications Taken**	0.969	0.944 to 0.995	0.009

Notes

a. Odds ratio predict the likelihood of being adherent to treatment. If odds ratio greater than one, it means a higher likelihood of adherence, thus a beneficial factor. On the contrary, odds ratio less than one means a harmful factor.

b. Income level is divided based on quintiles of zipcode level median household annual income (High: > = $50,307; Middle high: $38,576-$50,307; Middle low: $30,520-$38,576; Low: <$30,520)

### Persistence

**Fig 2:**
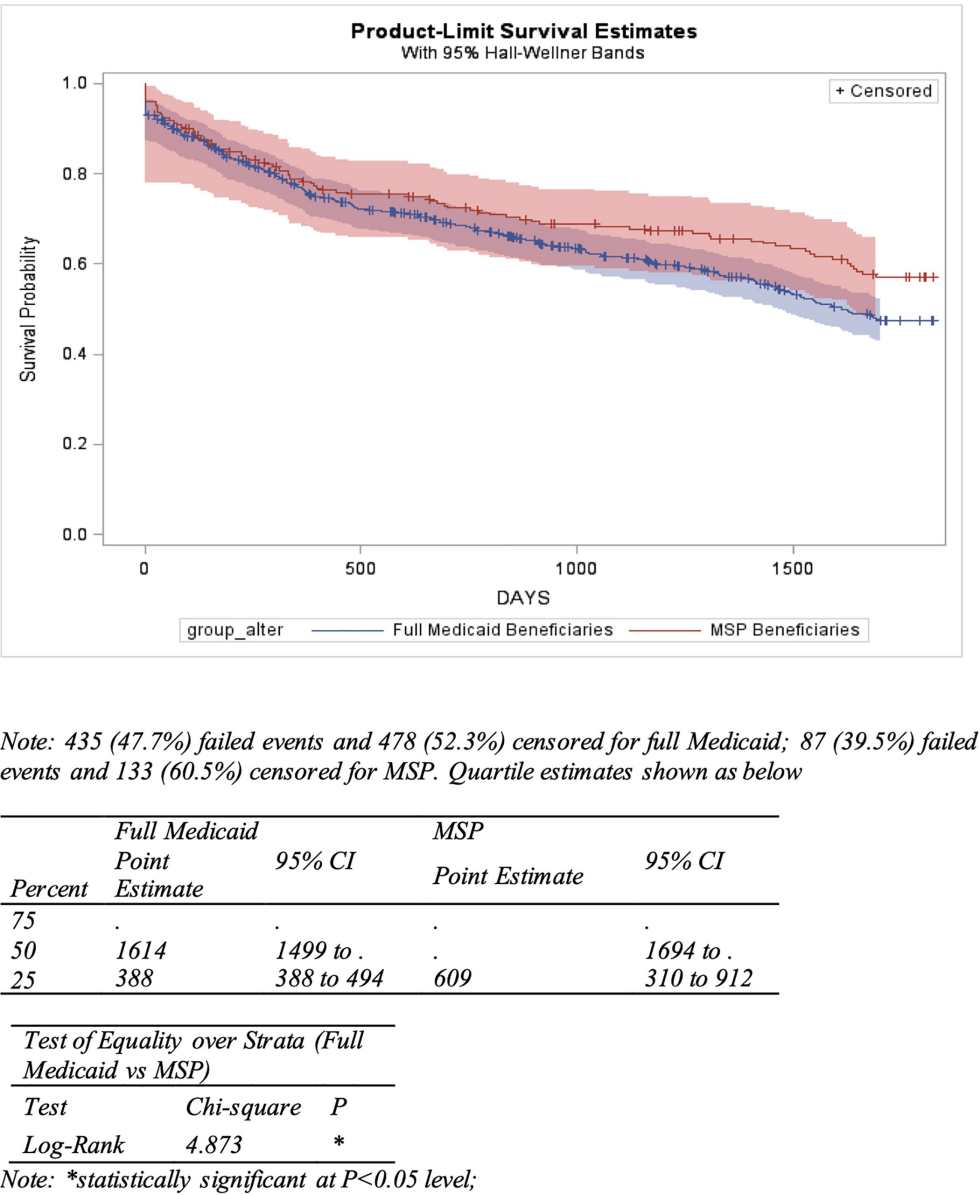
Kaplan Meier curve for the fraction of patients without a 180-day gap between two prescriptions fills since initiation—stratified by full Medicaid and MSP beneficiaries.

The unadjusted percentages of full Medicaid and MSP Beneficiaries that were persistent for Years 1 through 5 are shown in S5 Table. Based on both 180- and 90-day gap specifications, there were no statistically significant differences in persistence between the two treatment groups in all years. The Kaplan-Meier product-limit survival curves showing the fraction of patients remaining persistent (using 90-day gaps definition) as of a given time (in days after first prescription), stratified by treatment and control groups. The figure indicates that MSP beneficiaries are more likely to remain persistent compared with full Medicaid beneficiaries. Log rank test confirmed that for the 180-day specifications, the differences were large enough to be significant (Chisq: 4.873, p<0.05) (Fig 2). Under 180-day specifications MSP beneficiaries are also more likely to remain persistent compared with full Medicaid beneficiaries. However, log rank test showed that the difference was not significant (S2 Fig).

After adjustment for other factors, full Medicaid beneficiaries were not significantly different from MSP beneficiaries with respect to persistence (90-day gap). However, higher persistence was highly significantly associated with number of months with catastrophic insurance coverage, and lower persistence was significantly associated with total number of other medications taken. All other covariates were not significant. The model-based adjusted odds ratio of predictors for estimating the likelihoods of AI non-persistence based on 180-day gaps can be found in S6 Table.

## Discussion

Our study is the first of its kind to reveal the impact of small copayment differences on AHT adherence and persistence among the dual eligible breast cancer patients. Our study found that on average full Medicaid beneficiaries spent $1-$3 less per copayment than MSP beneficiaries on their standardized 30-day AI prescriptions. In our longitudinal hierarchical model predicting adherence, after adjustment for the other factors in the model, copayment reduction was not associated with a significantly better adherence, which was not surprising given the small amount of copayment differences. On the other hand, beneficiaries in both cohorts who reached the threshold for catastrophic coverage and had their copayments reduced to zero (which was on average a $1-$3 per copayment reduction for full Medicaid beneficiaries, $3-$5 per copayment reduction for MSP beneficiaries), were associated with significant improvement. Similar findings were reported in a previous study on Medicaid beneficiaries, where they found that even very small copayments caused Medicaid beneficiaries to delay or forgo medications [19]. In national surveys, respondents with incomes between 100%-200% federal poverty line had higher rates of cost-related medication underuse, food insecurity or both [27, 28]. When patients had to choose whether to spend their last dollars on food or medications, they were more likely to choose food and deter the medications they needed.

Common to all analyses is that we could not confirm the hypothesis that full Medicaid beneficiaries with lower copays would remain persistent longer than MSP beneficiaries. This result could be because full Medicare beneficiaries often still had the barrier of some copayment, or the apparent lack of difference could be due to a combination of unobserved environmental and personal factors, including different state policies on copayment collection, geographical locations on the distance from the nearest pharmacy, and the availability of mailed prescriptions [29, 30]. It is also worth noting that a patient may be adherent again in subsequent years, even after being non-persistent. Medication use after the first extended gap in prescriptions is not captured by the survival model.

After controlling for other covariates, race/ethnicity was not significantly associated with adherence or persistence. Our study could include only two race/ethnicity groups: non-white and white, non-Hispanic. Patients within the non-white group could not be further divided into more specific subgroups due to small sample size. It may be that race/ethnicity was not significantly associated with our outcomes, because our non-white group was composed of different subgroups with opposite associations with our outcomes. Previous studies found that controlling for covariates, Asian had higher adherence compared to white, while Black and Hispanic patients had lower [9, 21].

Our study has several strengths. First, our study filled gap in the literature by focusing on Medicare and Medicaid dual eligible beneficiaries, a commonly acknowledged as the most costly and usually financially vulnerable older patients with cancer in the U.S. [13, 14]. We examined the differential effects of copayment reduction and elimination on their AHT adherence. Second, our study was able to follow patients for their full course of AI treatment. Since AIs are the newer generation of AHT, which are gradually replacing tamoxifen for postmenopausal breast cancer patients, the results should be clinical meaningful, and help us understand the factors that impact patients’ response to taking AIs long-term. Third, our study used advanced statistical methods to derive the most accurate estimates possible for the effects of type of Medicaid coverage on our two outcomes. These methods included PSM to minimize potential selection bias due to non-random assignment, and longitudinal hierarchical modeling to control for correlated data within patient. In addition, we were able to accurately identify HR-positive early stage breast cancer patients with the tumor characteristics information in the SEER cancer registry. Finally, we had the exact dates of breast cancer diagnosis and start of AI treatment, which allowed us to calculate MPR and adherence per year and measure length of persistence to the day.

This study has several limitations. First, our study was observational, which means there were confounding factors in the theoretical framework that couldn’t be controlled for with the available data, such as patients’ knowledge of the disease, attitude toward treatment, and the ease of getting medications. These factors could exert different impacts on our treatment and control groups, which may have biased our findings downwards. However, on the other hand, patients who were more adherent to their hormone therapy may yield a higher chance to reach catastrophic phase, which was found in previous literature [31]. This may have biased the difference between our treatment and control group upwards. Second, due to data limitation, we were unable to control for personal level income differences in our analysis. However, we included zip code level annual median income to serve as a proxy. Third, we used Medicare claims to calculate adherence and persistence. However the amount of prescriptions that a patient filled does not necessarily equal to the amount of medication the patient consumed. Future qualitative studies interviewing patients for their behavior would be meaningful to check that our method for calculating adherence and persistence is appropriate. Finally, MSP beneficiaries were more likely to reach the catastrophic threshold faster than full Medicaid beneficiaries, given that they have on average a higher OOPC. Thus the effect of number of month in catastrophic phase may correlate with the difference between treatment and control groups. Our findings only suggested that patients who reached the threshold for catastrophic coverage were associated with significant improvement in adherence, however it doesn’t imply causality. One should interpret our findings with caution.

### Conclusions

This study used the features of full Medicaid and MSP benefits as an opportunity for a natural experiment to compare the impact of different levels of copayment reduction on AHT adherence and persistence among older patients with breast cancer. We found that reducing copayments on average from $4 to $2 did not find a significant impact on breast cancer patients’ adherence to their hormone therapy, but the elimination of copayment all together was associated with improved adherence and persistence. Previous literatures that compared to elimination of copayment found that even a minimal amount of cost sharing would inversely affect Medicaid patients’ medication treatment adherence and persistence [17–19]. Our results supported the findings from previous literature, however given previous mentioned limitations, our results should be interpreted with caution. Future legislative and advocacy efforts should be paid on eliminating cost sharing for Medicaid and Medicare dual eligible beneficiaries, and possibly even a broader group of financially vulnerable patients. Our study generated evidence on the separate effects of copayment reduction and elimination on drug adherence among breast cancer patients, which could be informative for potential expanding value-based insurance design plans to such field.

## Supporting information

S1 Fig Standardized variable differences plots for full Medicaid and MSP beneficiaries post to propensity scoring matching. (DOCX)

S2 Fig Kaplan Meier curve for the fraction of patients without a 180-day gap between two prescriptions fills since initiation—stratified by full Medicaid and MSP beneficiaries. (DOCX)

S1 Table Descriptions of variables. (DOCX)

S2 Table Average standardized 30-day out-of-pocket costs for full Medicaid and MSP beneficiaries by year (year 1 to year 5). (DOCX)

S3 Table Percentage of dual eligible beneficiaries were adherent to hormone therapy and average medication procession ratio (MPR) from year 1 to year 5, by treatment and control groups. (DOCX)

S4 Table Model-based adjusted AI adherence measured by medication possession ratio (%) among Medicare and Medicaid dual eligible beneficiaries diagnosed with hormone receptor-positive early stage breast cancer from 2007 to mid-2009. (DOCX)

S5 Table Unadjusted percentage of dual eligible beneficiaries were persistent (no more than 90-, and 180-day gaps between two filled prescriptions) to hormone therapy from year 1 to year 5, by treatment and control groups. (DOCX)

S6 Table Model-based adjusted odds ratio of predictors for AI non-persistence (at least one 90-, and 180-day gaps) among women diagnosed with hormone receptor-positive early stage breast cancer from 2007 to mid-2009. (DOCX)
