# Supplementary material for: Cost sharing for breast cancer hormone therapy: How do dual eligible patients’ copayment impact adherence
Source: PLoS One. 2021 May 18;16(5):e0250967. doi: 10.1371/journal.pone.0250967 (PMC8130966; doi:10.1371/journal.pone.0250967)
Supplement: S1 Fig — (DOCX) [file pone.0250967.s001.docx]

*S1 Fig. Standardized Variable Differences Plots for Full Medicaid and MSP Beneficiaries post to Propensity Scoring Matching*

*
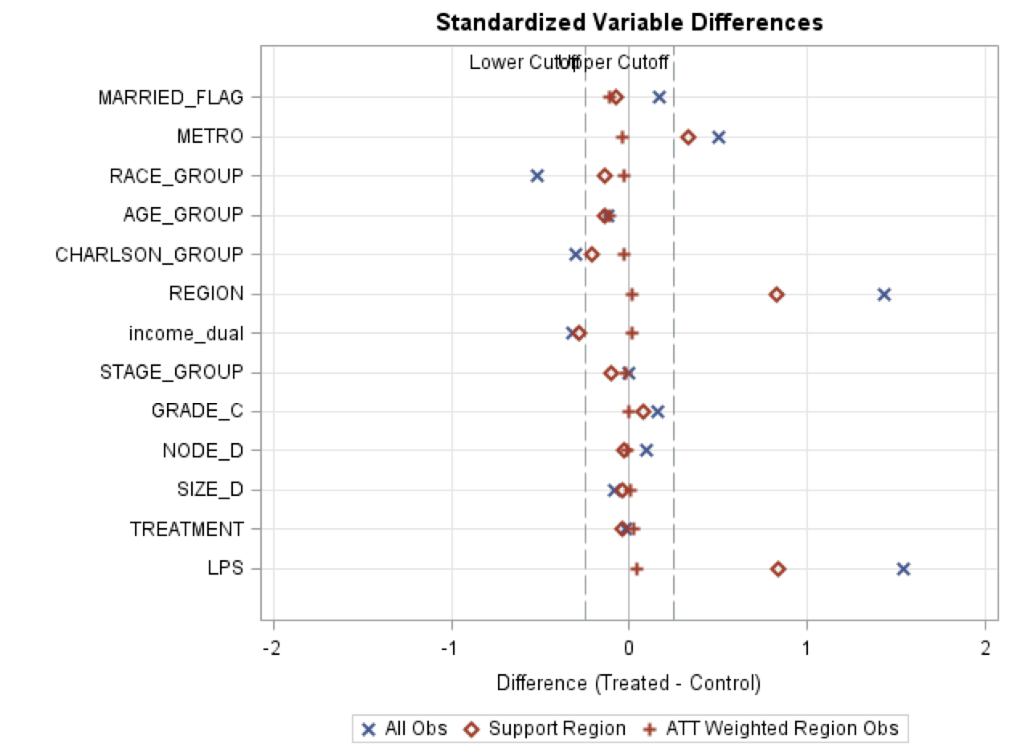
*
