# Supplementary material for: Cost sharing for breast cancer hormone therapy: How do dual eligible patients’ copayment impact adherence
Source: PLoS One. 2021 May 18;16(5):e0250967. doi: 10.1371/journal.pone.0250967 (PMC8130966; doi:10.1371/journal.pone.0250967)
Supplement: S2 Fig — (DOCX) [file pone.0250967.s002.docx]

*S2 Fig. Kaplan Meier Curve for the Fraction of Patients Without a 180-day Gap between Two Prescriptions Fills since Initiation - Stratified by Full Medicaid and MSP Beneficiaries*


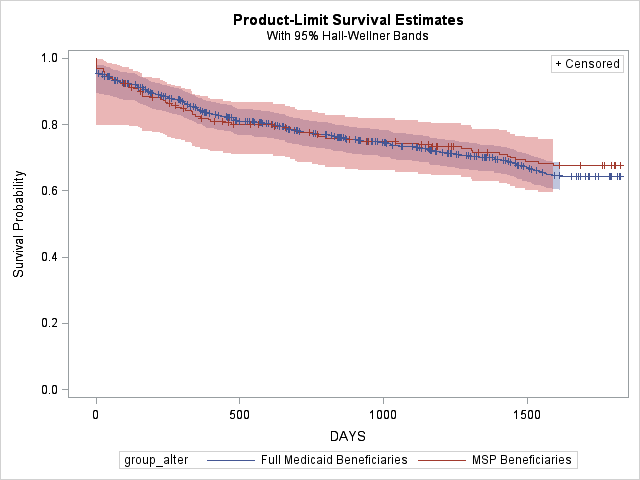


*Note: 293 (32.1%) failed events and 620 (67.9%) censored for full Medicaid; 66 (30.0%) failed events and 154 (70.0%) censored for MSP. Quartile estimates shown as below*

|  | *Full Medicaid* | | *MSP* | |
| --- | --- | --- | --- | --- |
| *Percent* | *Point Estimate* | *95% CI* | *Point Estimate* | *95% CI* |
| *75* | *.* | *.* | *.* | *.* |
| *50* | *.* | *.* | *.* | *.* |
| *25* | *955* | *716 to 1165* | *912* | *441 to 1495* |
