# Supplementary material for: Cost sharing for breast cancer hormone therapy: How do dual eligible patients’ copayment impact adherence
Source: PLoS One. 2021 May 18;16(5):e0250967. doi: 10.1371/journal.pone.0250967 (PMC8130966; doi:10.1371/journal.pone.0250967)
Supplement: S1 Table — (DOCX) [file pone.0250967.s003.docx]

*S1 Table. Descriptions of Variables*

| **VARIABLE NAME** | | **DEFINITION** |
| --- | --- | --- |
| **DEPENDENT VARIABLES** | |  |
| Adherence continuous | | A continuous variable of MPR % |
| Adherence dummy | | A dummy equal to 1 if MPR >=80% |
| Persistence continuous | | A continuous variable of number of gap days |
| Persistence dummy | | A dummy equal to 1 if gap < 60, 90, or 180 days |
|  | |  |
| **TREATMENT VARIABLES** | |  |
| Catastrophic coverage months | | A continuous variable of number of months under catastrophic coverage |
| Full Medicaid beneficiaries | | A dummy equal to 1 if full Medicaid beneficiaries |
|  | |  |
| **CONTROL VARIABLES** | |  |
| Race/Ethnicity | | A dummy variable equal to 1 if White, non-Hispanic |
| Age continuous | | A continuous variable, 65+ years old |
| Married | | A dummy variable equal to 1 if married |
| Income level | | A categorical variable where  1 Low (median: <25%)  2 Middle low (median: 25-50%)  3 Middle high (median: 50-75%)  4 High (median: >75%) |
| Metropolitan area* | | A dummy variable equal to 1 if big metro or metro |
| Tumor Stage | | A categorical variable where  1 Stage I  2 Stage II  3 Stage III |
| Tumor Size (cm) | | A categorical variable where  1 <1.0  2 >= 1.0  3 unknown |
| Number of Positive Nodes | | A categorical variable where  1 0 (node negative)  2 >=1  3 unknown |
| (continued the next page) | | |
| Tumor Grade | | A categorical variable where  1 Well differentiated  2 Moderately differentiated  3 Poorly differentiated  4 unknown |
| Initial Surgery/Radiation Treatment | | A categorical variable where  1 No surgery  2 Surgery (breast-conserving surgery or mastectomy) + radiation  3 Surgery, no radiation |
| Comorbidity Score  (see detailed construction description on NCI website: <https://healthcaredelivery.cancer.gov/>  seermedicare/considerations/comorbidity.html) | | A categorical variable where  1 0  2 1  3 2  4 3+ |
| Number of Medication | A continuous variable of number of medications a patient takes each year | |
