# Supplementary material for: Cost sharing for breast cancer hormone therapy: How do dual eligible patients’ copayment impact adherence
Source: PLoS One. 2021 May 18;16(5):e0250967. doi: 10.1371/journal.pone.0250967 (PMC8130966; doi:10.1371/journal.pone.0250967)
Supplement: S2 Table — (DOCX) [file pone.0250967.s004.docx]

*S2 Table. Average Standardized 30-day Out-of-pocket Costs for Full Medicaid and MSP Beneficiaries by Year (Year 1 to Year 5)*

|  | |  | **n** | **Mean, $** | **Median, $** | **Min, $** | **p25, $** | **p75, $** | **Max, $** |
| --- | --- | --- | --- | --- | --- | --- | --- | --- | --- |
| **Year1** | | Full Medicaid | 220 | 2.00 | 2.26 | 0.00 | 1.03 | 3.10 | 11.27 |
|  | | MSP | 913 | 4.77 | 5.44 | 0.00 | 3.62 | 5.80 | 17.00 |
| **Year2** | | Full Medicaid | 210 | 1.46 | 1.20 | 0.00 | 0.00 | 2.50 | 6.27 |
|  | | MSP | 858 | 3.27 | 3.50 | 0.00 | 1.47 | 5.60 | 6.30 |
| **Year3** | | Full Medicaid | 194 | 0.98 | 0.76 | 0.00 | 0.00 | 1.50 | 5.52 |
|  | | MSP | 801 | 2.42 | 2.26 | 0.00 | 0.83 | 3.60 | 6.87 |
| **Year4** | | Full Medicaid | 185 | 0.64 | 0.38 | 0.00 | 0.00 | 1.10 | 3.42 |
|  | | MSP | 737 | 1.70 | 1.78 | 0.00 | 0.48 | 2.54 | 6.87 |
| **Year5** | | Full Medicaid | 167 | 0.50 | 0.38 | 0.00 | 0.00 | 1.01 | 3.56 |
|  | | MSP | 671 | 1.37 | 1.05 | 0.00 | 0.00 | 2.55 | 6.50 |
|  |  | | | |  |  |  |  |  |
