# Supplementary material for: Cost sharing for breast cancer hormone therapy: How do dual eligible patients’ copayment impact adherence
Source: PLoS One. 2021 May 18;16(5):e0250967. doi: 10.1371/journal.pone.0250967 (PMC8130966; doi:10.1371/journal.pone.0250967)
Supplement: S3 Table — (DOCX) [file pone.0250967.s005.docx]

*S3 Table. Percentage of Dual Eligible Beneficiaries Were Adherent to Hormone Therapy and Average Medication Procession Ratio (MPR) from Year 1 to Year 5, by Treatment and Control Groups*

|  | **Adherent by year** | | |  | **MPR by year** | | | |
| --- | --- | --- | --- | --- | --- | --- | --- | --- |
|  | **Full Medicaid** | **MSP** | **P** |  | | **Full Medicaid** | **MSP** | **P** |
| First Year n (%) | 178 (80.9) | 726 (79.5) | NS | First Year Mean, % | | 86.8 | 86.8 | NS |
| Second Year n (%) | 147 (70.0) | 598 (69.7) | NS | Second Year Mean, % | | 74.3 | 76.1 | NS |
| Third Year n (%) | 140 (72.2) | 518 (64.7) | * | Third Year Mean, % | | 73.7 | 72.4 | NS |
| Fourth Year n (%) | 126 (68.1) | 467 (63.4) | NS | Fourth Year Mean, % | | 71.3 | 69.4 | NS |
| Fifth Year n (%) | 94 (56.3) | 357 (53.2) | NS | Fifth Year Mean, % | | 64.5 | 62.7 | NS |

*Note: *statistically significant at p<0.05 level, ** at p<0.01 level, *** at p<0.001 level;*

*NS stands for not significant*
