# Supplementary material for: Cost sharing for breast cancer hormone therapy: How do dual eligible patients’ copayment impact adherence
Source: PLoS One. 2021 May 18;16(5):e0250967. doi: 10.1371/journal.pone.0250967 (PMC8130966; doi:10.1371/journal.pone.0250967)
Supplement: S4 Table — (DOCX) [file pone.0250967.s006.docx]

*S4 Table. Model-based Adjusted AI Adherence Measured by Medication Possession Ratio (%) Among Medicare and Medicaid Dual Eligible Beneficiaries Diagnosed with Hormone Receptor-Positive Early Stage Breast Cancer from 2007 to Mid-2009*

| **Variable** | **Estimates** | **P** |
| --- | --- | --- |
| **Treatment and Control** |  |  |
| Full Medicaid vs MSP | 0.037 | NS |
| **Catastrophic Coverage Months** | 0.038 | *** |
| **Year** |  |  |
| 2 vs 1 | -0.128 | *** |
| 3 vs 1 | -0.150 | *** |
| 4 vs 1 | -0.147 | *** |
| 5 vs 1 | -0.226 | *** |
| **Age, y** | -0.005 | NS |
| **Race** |  |  |
| Non-White vs White, non-Hispanic | 0.020 | NS |
| **Comorbidity Score** |  |  |
| 1 vs 0 | -0.014 | NS |
| 2 vs 0 | -0.020 | NS |
| 3+ vs 0 | -0.064 | NS |
| **Married** |  |  |
| Yes vs No | -0.012 | NS |
| **Income level** |  |  |
| High vs Low | 0.042 | * |
| Middle high vs Low | -0.015 | NS |
| Middle low vs Low | -0.025 | NS |
| **SEER Registry Region** |  |  |
| Midwest vs West | 0.055 | NS |
| Northeast vs West | 0.066 | NS |
| South vs West | 0.017 | NS |
| **Metropolitan Area** |  |  |
| Yes vs No | 0.001 | NS |
| **Tumor Stage** |  |  |
| II vs I | 0.018 | NS |
| (continued the next page) | | |
| **Variable** | **Estimates** | **P** |
| III vs I | -0.034 | NS |
| **Lymph Node Positivity** |  |  |
| >=1 vs 0 (negative) | -0.011 | NS |
| **Tumor Size** |  |  |
| >=1cm vs <1cm | 0.015 | NS |
| **Tumor Grade** |  |  |
| Moderately vs Well differentiated | 0.008 | NS |
| Poorly vs Well differentiated | 0.024 | NS |
| **Treatment** |  |  |
| Surgery + radiation vs No surgery | 0.121 | NS |
| Surgery, no radiation vs No surgery | 0.100 | NS |
| **Number of Medications Taken** | 0.005 | * |

*Note: *statistically significant at p<0.05 level, ** at p<0.01 level,*

**** at p<0.001 level; NS stands for not significant*
