# Supplementary material for: Cost sharing for breast cancer hormone therapy: How do dual eligible patients’ copayment impact adherence
Source: PLoS One. 2021 May 18;16(5):e0250967. doi: 10.1371/journal.pone.0250967 (PMC8130966; doi:10.1371/journal.pone.0250967)
Supplement: S5 Table — (DOCX) [file pone.0250967.s007.docx]

*S5 Table. Unadjusted Percentage of Dual Eligible Beneficiaries Were Persistent (No More Than 90-, and 180-day Gaps Between Two Filled Prescriptions) to Hormone Therapy from Year 1 to Year 5, by Treatment and Control Groups*

|  | **90-day gaps** | | | **180-day gaps** | | |
| --- | --- | --- | --- | --- | --- | --- |
|  | **Full Medicaid** | **MSP** | **P** | **Full Medicaid** | **MSP** | **P** |
| First Year n (%) | 173 (78.6) | 704 (77.1) | NS | 181 (82.3) | 773 (84.7) | NS |
| Second Year n (%) | 152 (92.7) | 596 (89.5) | NS | 163 (94.8) | 672 (92.2) | NS |
| Third Year n (%) | 137 (94.5) | 509 (90.4) | NS | 147 (96.1) | 592 (94.7) | NS |
| Fourth Year n (%) | 123 (93.9) | 421 (89.4) | NS | 132 (93.6) | 513 (93.4) | NS |
| Fifth Year n (%) | 102 (89.5) | 341 (86.8) | NS | 118 (97.5) | 444 (94.3) | NS |

*Note: *statistically significant at p<0.05 level, ** at p<0.01 level, *** at p<0.001 level; NS stands for not significant*
