# Supplementary material for: Cost sharing for breast cancer hormone therapy: How do dual eligible patients’ copayment impact adherence
Source: PLoS One. 2021 May 18;16(5):e0250967. doi: 10.1371/journal.pone.0250967 (PMC8130966; doi:10.1371/journal.pone.0250967)
Supplement: S6 Table — (DOCX) [file pone.0250967.s008.docx]

*S6 Table. Model-based Adjusted Odds Ratio of Predictors for AI Non-Persistence (At Least One 90-, and 180-day Gaps) Among Women Diagnosed with Hormone Receptor-Positive Early Stage Breast Cancer From 2007 to Mid-2009*

|  | **90-day gaps** | | | | **180-day gaps** | | | |  |  |
| --- | --- | --- | --- | --- | --- | --- | --- | --- | --- | --- |
| **Variable** | **Odds Ratio** | | **P** | | **Odds Ratio** | | **P** | |  |  |
| **Treatment and Control** |  | |  | |  | |  | |  |  |
| Full Medicaid vs MSP | 0.858 | |  | | 1.029 | |  | |  |  |
| **Catastrophic Coverage Months** | 0.752 | | *** | | 0.701 | | *** | |  |  |
| **Age, y** | 0.997 | |  | | 1.008 | |  | |  |  |
| **Race/Ethnicity** |  | |  | |  | |  | |  |  |
| Non-White vs White, non-Hispanic | 0.923 | |  | | 0.933 | |  | |  |  |
| **Comorbidity score** | |  | |  | |  | |  | |  |
| 1 vs 0 | 1.109 | |  | | 1.036 | |  | |  |  |
| 2 vs 0 | 1.042 | |  | | 0.971 | |  | |  |  |
| 3+ vs 0 | 1.042 | |  | | 0.859 | |  | |  |  |
| **Married** |  | |  | |  | |  | |  |  |
| Yes vs No | 0.941 | |  | | 0.867 | |  | |  |  |
| **Income level** |  | |  | |  | |  | |  |  |
| High vs Low | 0.606 | |  | | 0.842 | |  | |  |  |
| Middle high vs Low | 0.867 | |  | | 0.890 | |  | |  |  |
| Middle low vs Low | 0.986 | |  | | 1.113 | |  | |  |  |
| **SEER Registry Region** | |  | |  | |  | |  | |  |
| Midwest vs West | 0.977 | |  | | 0.794 | |  | |  |  |
| Northeast vs West | 1.069 | |  | | 0.763 | |  | |  |  |
| South vs West | 0.908 | |  | | 0.803 | |  | |  |  |
| **Metropolitan Area** |  | |  | |  | |  | |  |  |
| Yes vs No | 0.929 | |  | | 0.873 | |  | |  |  |
| **Tumor Stage** |  | |  | |  | |  | |  |  |
| II vs I | 0.868 | |  | | 0.613 | |  | |  |  |
| III vs I | 1.158 | |  | | 0.101 | |  | |  |  |
| **Lymph Node Positivity** | |  | |  | |  | |  | |  |
| >=1 vs 0 (negative) | 1.117 | |  | | 1.243 | |  | |  |  |
| **Tumor Size** |  | |  | |  | |  | |  |  |
| (continued the next page) | | | | | | | | |  |  |
|  | **90-day gaps** | | | | **180-day gaps** | | | |  |  |
| **Variable** | **Odds Ratio** | | **P** | | **Odds Ratio** | | **P** | |  |  |
| >1cm vs <1cm | 0.952 | |  | | 1.029 | |  | |  |  |
| **Tumor Grade** |  |  | |  | |  | |  | |  |
| Moderately vs well differentiated | 0.930 | |  | | 0.855 | |  | |  |  |
| Poorly vs well differentiated | 1.163 | |  | | 1.061 | |  | |  |  |
| **Treatment** |  | |  | |  | |  | |  |  |
| Surgery + radiation vs no surgery | 0.888 | |  | | 0.784 | |  | |  |  |
| Surgery, no radiation vs no surgery | 0.841 | |  | | 0.862 | |  | |  |  |
| **Number of Medication Taken** | 1.066 | | *** | | 1.059 | | * | |  |  |

*Notes:*

*a. Odds ratio predict the likelihood of a non-persistence event. If odds ratio greater than one, it means a higher likelihood of non-persistence, thus a harmful effect. On the contrary, odds ratio less than one means a beneficial effect.*

*b. *statistically significant at P<0.05 level, ** at P<0.01 level, *** at P<0.001 level; Blank P value means not statistically significant*
